# Fluorophore-conjugated 4-1BB antibody enables early detection of T-cell responses in inflammatory arthritis via NIRF imaging

**DOI:** 10.1007/s00259-022-05946-y

**Published:** 2022-09-07

**Authors:** Shao Duan, Chaozhe Han, Yifei Xia, Chengwei Jing, Bohan Dong, Xin Zhang, WeiWei Wang, Yu Wang, Maomao Zhang, Ping Li, Weiyu Chen, Zunyu Xiao, Chao Li

**Affiliations:** 1 Department of Orthopedics, the Second Affiliated Hospital of Harbin Medical University, Harbin, China; 2 First Affiliated Hospital, Heilongjiang University of Chinese Medicine, Harbin, China; 3 Key Laboratory of Myocardial Ischemia, Chinese Ministry of Education, Harbin, China; 4 Department of Nephrology, the Second Affiliated Hospital of Harbin Medical University Harbin, Harbin, China; 5 Department of Radiology and Nuclear Medicine, the Second Affiliated Hospital of Harbin Medical University, Harbin, China; 6 International Institutes of Medicine, The Fourth Affiliated Hospital, Zhejiang University School of Medicine, Hangzhou, China; 7 Molecular Imaging Research Center of Harbin Medical University, Harbin, China

**Keywords:** CD137/4-1BB (Tumour necrosis factor receptor superfamily 9), Activated T cells, Inflammatory arthritis, NIRF imaging

## Abstract

**Purpose:**

We first developed a 4-1BB-targeted optical probe, named IRDye-680RD-4-1BB mAb (monoclonal antibody), and evaluated its value for the detection of 4-1BB^+^ activated T cells in vivo as well as the diagnosis of rheumatoid arthritis (RA) in an adjuvant-induced arthritis (AIA) mouse model.

**Methods:**

The 4-1BB expression pattern was analysed by flow cytometry and immunofluorescence (IF) staining. The 4-1BB mAb was conjugated with IRDye-680RD NHS ester, and characterized via fluorescence spectrum. A cell-binding assay was also performed to assess the interaction of this probe with activated and naïve murine T cells. Longitudinal near-infrared fluorescence (NIRF) imaging of the probe was performed at 6, 24, 48, 72, and 96 h after probe administration.

**Results:**

4-1BB expression was highly upregulated during the pathogenesis of RA. Good colocalization was also observed between CD3 and 4-1BB by IF staining and t-SNE (T-distributed stochastic neighbour embedding) analysis, which indicates that 4-1BB was mainly expressed on T cells. Compared to the control group, a significantly higher signal was observed in the right hind paw (RP) of mice with AIA at all time points. The ex vivo biodistribution study results were consistent with the in vivo NIRF imaging results, which validated the accuracy of the region of interest (ROI) measurements. The sensitivity against 100% specificity observed in the receiver operator characteristic (ROC) curve analysis could distinguish the AIA group from the control group at all time points, indicating the value of IRDye-680RD-4-1BB mAb for RA diagnosis.

**Conclusion:**

We successfully developed a novel optical imaging probe, named IRDye-680RD-4-1BB mAb, for tracking 4-1BB^+^ activated T cells in vivo, and 4-1BB NIRF imaging is a promising strategy for noninvasively detecting the pathogenesis of RA.

**Supplementary Information:**

The online version contains supplementary material available at 10.1007/s00259-022-05946-y.

## Introduction

Rheumatoid arthritis (RA) is a chronic inflammatory autoimmune disease that usually causes bone damage, loss of joint function, and other complications, such as heart disease and diabetes, at later stages [1]. Precise diagnosis and timely intervention are keys for optimizing the whole therapeutic process, which may lead to better prognosis for RA patients in the clinic [2, 3]. Given their low specificity and sensitivity, current approaches such as measuring rheumatoid factor (RF) and cyclic citrullinated peptide (CCP) levels in blood [2], as well as anatomic imaging, often fail in early diagnosis of RA [4]. Thus, there is an urgent need to develop reliable tools to achieve this goal.

Inappropriate T-cell activation and imbalanced T cell–related immune responses are critical mediators of the pathogenesis of RA [5], and the detection of activated T cells via noninvasive molecular imaging theoretically represents an ideal solution for the early diagnosis of RA. The most classic positron emission tomography (PET) tracer, 2-deoxy-2-^18^F-fluoro-D-glucose (^18^F-FDG), has been tested in many studies [6–8]. Despite the achievements of ^18^F-FDG, the carbohydrate metabolic pathway is also active in many other types of cells and tissues [9], which definitely causes false-positive results. In a recent study, an analogue of arabinose guanine (AraG), 2′-deoxy-2′-^18^F-fluoro-9-β-D-arabinofuranosylguanine (^18^F-AraG), was used to detect activated T cells in an adjuvant-induced arthritis (AIA) model [10]. However, ^18^F-AraG was also uptake by macrophages, dendritic cells (DCs) and activated B cells in the inflammatory environment of RA [11]. To overcome these challenges, our group developed PET probes targeting endogenous T-cell costimulatory biomarkers, which enables detection of activated T cells in vivo with high specificity and sensitivity. The benefit of these PET probes is that we can precisely predict and monitor the therapeutic effects of cancer immunotherapy [12], and the utility of these imaging techniques enables early diagnosis of T cell–mediated inflammatory diseases [13] prior to the onset of clinical symptoms. Thus, the detection of costimulatory molecules on activated T cells may potentially overcome the current difficulties in the early diagnosis of RA.

4-1BB (CD137, tumour necrosis factor receptor superfamily 9) is an inducible costimulatory receptor that is mainly expressed on activated CD4^+^ and CD8^+^ T cells [14]. After 4-1BB/4-1BBL ligation, the downstream costimulatory signal triggers T-cell expansion, cytokine production (interferon-γ and interleukin-2) and enhanced effector function [15]. Benefiting from the RNA sequencing dataset, we previously validated inducible T-cell costimulatory receptor (ICOS) as one of the most ideal biomarkers for the PET imaging of CAR-T cells. Using a similar approach, in the current study, we identified 4-1BB as a novel imaging biomarker for tracking activated T cells in an inflammatory arthritis mouse model. The expression pattern of 4-1BB at different time points in mice with AIA was first confirmed via flow cytometry and IF staining. Then, an IRDye-680RD-conjugated 4-1BB mAb, named IRDye-680RD-4-1BB mAb, was used for NIRF imaging study. Overall, we have demonstrated that IRDye-680RD-4-1BB mAb is a NIRF imaging probe that can be used to detect activated T cells in vivo, and moreover, NIRF imaging targeting 4-1BB is a promising approach for diagnosing inflammatory arthritis.

## Materials and methods

### General reagents

InVivo MAb anti-mouse 4-1BB (clone: 3H3) and InVivoPlus rat IgG2a isotype control (clone: 2A3) were supplied by BioXCell (New Hampshire, USA). IRDye® 680RD NHS Ester Infrared Dye was supplied by Li-cor (Nebraska, USA). Fixable Viability Stain 780 was purchased from BD Pharmingen (San Diego, USA). Dimethyl sulfoxide (DMSO) Hybri-Max (TM) sterile-filtered BioReagent was purchased from Sigma (St. Louis, MO, USA). The AbC™ total antibody compensation bead kit was acquired from Thermo Fisher (Waltham, MA, USA). PE anti-mouse CD4, FITC anti-mouse CD8a, APC anti-mouse CD137, and Alexa Fluor 594 goat anti-rat IgG (minimal x-reactivity) antibodies were supplied by BioLegend (San Diego, CA, USA). Anti-rabbit IgG (H + L), F(ab')2 fragment (Alexa Fluor® 488 Conjugate) was purchased from Cell Signaling Technology (Massachusetts, USA). A purified anti-mouse CD3 antibody was supplied by Thermo (Waltham, MA, USA). An anti-CD137 antibody was purchased from Abcam (Cambridgeshire, UK).

### Library construction for RNA-seq and sequencing procedures

Total RNA was isolated using a RNeasy Mini Kit (Qiagen, Hilden, Germany). Paired-end libraries were synthesized by using the TruSeq® RNA Sample Preparation Kit (Illumina, CA, USA) following the TruSeq® RNA Sample Preparation Guide. Briefly, poly-A-containing mRNA molecules were purified using poly-T oligo-attached magnetic beads. After purification, the mRNA was fragmented into small pieces using divalent cations at 94 °C for 8 min. The cleaved RNA fragments were synthesized into first-strand cDNA using reverse transcriptase and random primers. This was followed by second-strand cDNA synthesis using DNA Polymerase I and RNase H. These cDNA fragments were then subjected to an end repair process, the addition of a single ‘A’ base, and then ligation of the adapters. The products were purified and enriched by PCR to create the final cDNA library. Purified libraries were quantified by Qubit® 2.0 Fluorometer (Life Technologies, MD, USA) and validated by an Agilent 2100 bioanalyzer (Agilent Technologies, CA, USA) to confirm the insert size and calculate the molar concentration. The cluster was generated by cBot with the library diluted to 10 pM and then sequenced on the Illumina HiSeq Xten (Illumina, CA, USA). Library construction and sequencing were performed at Shanghai Biotechnology Corporation (Shanghai, China).

### Fluorophore-conjugation IRDye-680RD to 4-1BB mAb

IRDye-680RD NHS ester was dissolved in DMSO to a final concentration of 5 mg/mL. The 4-1BB mAb was diluted to 1 mg/mL with sterile PBS (phosphate-buffered saline). Furthermore, IRDye-680RD NHS ester was added to 4-1BB mAb, and the molar ratio of 4-1BB mAb to IRDye-680RD was 1:10. The mixture was incubated overnight at 4 °C. A Vivaspin2 50 kDa MWCO cut-off spin filter (GE Healthcare, New Jersey, USA) was used for purification. The final concentration of the IRDye-680RD-4-1BB mAb was measured by a NanoDrop 2000 UV − vis spectrophotometer (Thermo Fisher, Waltham, MA, USA).

### LC‒MS/MS analysis

Liquid Chromatograph Triple Quadrupole Mass Spectrometer (LC‒MS/MS) analysis was performed according to the following protocols. An appropriate amount of protein solution was placed into a 1.5 ml EP tube, which was centrifuged at 12,000 rcf for 10 min at 4 °C, and the supernatant was collected. We used an Ultimate 3000 (Thermo Fisher Scientific, USA) column: ACQUITY UPLC Protein BEH C4 Column (300 Å, 1.7 µm, 2.1 mm × 50 mm) with the following parameters: mobile phase: A: 0.1% formic acid in water; mobile phase B: 0.1% formic acid in acetonitrile; total flow rate: 0.300 ml/min; and LC linear gradient from 5 to 100% B for 10 min, from 100 to 100% B for 3 min, from 100 to 5% B for 1 min, and from 5 to 5% B for 1 min.

### T cell isolation, activation and cell uptake study

The BALB/c mice (Charles River Laboratories, MA, USA) were anaesthetized under 2% isoflurane. Immediately after euthanasia, spleens were collected and homogenized through a 40 μm cell strainer. T cells were isolated according to the standard protocol of the EasySep Mouse T-cell Isolation Kit (StemCell Technologies, Vancouver, BC, Canada). After isolation, the T cells were incubated with Cell Stimulation Cocktail (Thermo Fisher, USA) in Iscove’s Modified Dulbecco’s Medium (IMDM, Thermo Fisher, USA) at 37 °C. Seventy-two hours later, both resting and activated T cells were collected, counted, and incubated with IRDye-680RD-4-1BB mAb for 1 h at 37 °C. After 3 washes with sterile PBS (Sigma, St. Louis, MO, USA), all the samples were analysed using a BD flow cytometer (San Diego, USA).

### Establishment of the AIA mouse model

All the animal studies were conducted under protocols approved by the Local Ethical Committee of Harbin Medical University Animal Care and Use. An AIA mouse model was used for the in vivo optical imaging study. Typically, 8- to 10-week-old male BALB/c mice (20–25 g) received a single injection of 0.5 mL/kg complete Freund’s adjuvant (CFA, Chondrex, Inc., Woodinville, USA) into the plantar subcutaneous tissue of the right hind paw (affected paw [AP]) or an equal amount of sterile PBS injected into a control paw (control paw [CP]). To maintain inflammatory arthritis, 7 days after the first injection, a second dose was administered. The animals were monitored every other day, and the severity of arthritis in each paw was scored by visual inspection on a scale of 0 to 4 according to the degree of inflammation. A score of 0 indicates no evidence of erythema and swelling; a score of 1 indicates erythema in the tarsals or ankle joint; a score or 2 indicates erythema and mild swelling extending from the ankle to the tarsals; a score of 3 indicates erythema and moderate swelling extending from the ankle to metatarsal joints; and a score of 4 indicates erythema and severe swelling encompassing the ankle, foot, and digits. Paw swelling associated with arthritis severity was objectively determined by measuring paw thickness with a calliper.

### Enzyme-linked immunosorbent assay (ELISA)

Tissue samples were collected and weighed, PBS (pH 7.2–7.4) was added, and the samples were frozen with liquid nitrogen. After thawing at 2–8 °C, the samples were homogenized by hand or with grinders and centrifuged for 20 min at 2000–3000 rpm, and the supernatants were collected. ELISA was performed according to the standard protocol provided by MEIMIAN company (Jiang Su, China).

### FACS cytometry and IF staining

The mouse spleens and paws were harvested on Day 11. Single-cell suspensions were generated according to a previous protocol [16]. FACS staining was performed at 4 °C using the following antibodies: PE anti-mouse CD4, FITC anti-mouse CD8a, and APC anti-mouse CD137. Data were acquired on a BD FACSCanto II Flow Cytometer and analysed using FlowJo version 10.7.1.

For IF staining, the right paws were harvested on Day 8, fixed with 4% PFA (paraformaldehyde) for 24 h, and decalcified with 10% ETDA (Thermo, Waltham, MA, USA) for 20 days. The samples were embedded in paraffin and cut into 5-μm-think sections. CD3 and 4-1BB primary antibodies were incubated overnight at 4 °C. Secondary antibodies were incubated at room temperature for 1 h. The sections were finally stained with DAPI and imaged with confocal microscopy (Carl Zeiss, Jena, Germany).

### In vivo NIRF imaging and ex vivo biodistribution

In vivo fluorescence imaging was performed using a Bruker InVivo FX PRO system (Bruker, MA, USA), and the results were analysed using Bruker Molecular Imaging Software (IB5438150 Rev. B 12/12, Bruker, USA). Generally, mice were anaesthetized with 2% isoflurane and injected with 20 μg IRDye-680RD-4-1BB mAb through the tail vein. Images were acquired at 6, 24, 48, 72 and 96 h after probe injection, with the following settings: f-stop 2.5, FOV 200 mm, 750 nm WA Emission Filter, 72 mm, 670 nm Excitation Filter, 25 mm. For ex vivo imaging, immediately after the final scan, the mice were euthanized, and the organs (heart, liver, lung, spleen, kidney, right paw, left paw, femur, muscle and intestine) were collected and imaged under the same settings. All the data were analysed via Bruker Molecular Imaging Software. The quantified data were normalized as p/s/cm^2^/sr.

### Statistical analysis

All the data analysis was performed with PRISM 8 (GraphPad). One- or two-way ANOVA and unpaired 2-tailed Student’s *t* test were used for data analysis where appropriate. *P* values less than 0.05 were considered statistically significant.

## Results

### 4-1BB mRNA was highly upregulated upon RA onset

**Fig. 1:**
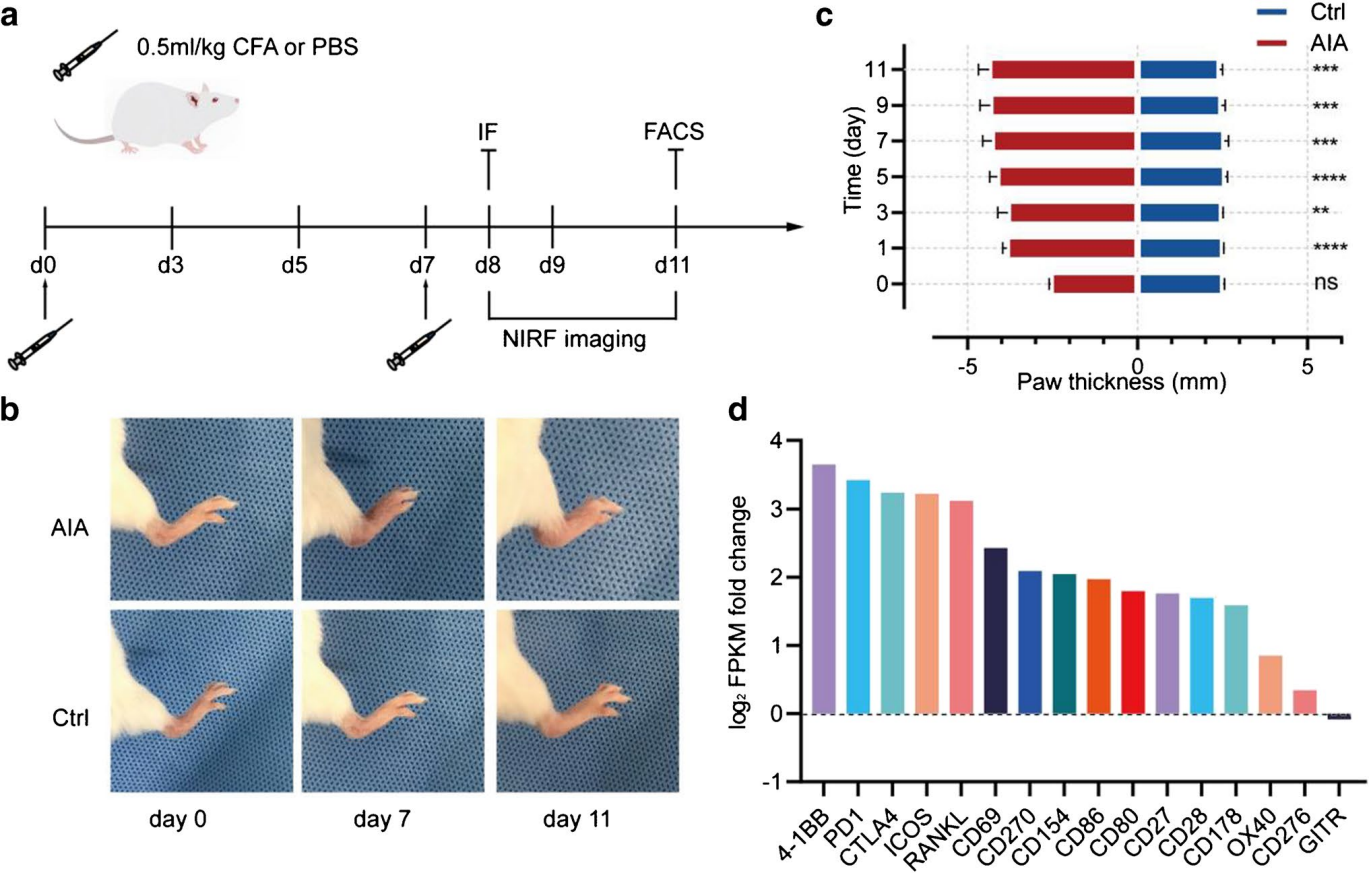
**a** Study design: 0.5 ml/kg CFA or PBS was injected into the right hind paw on Day 0 and 7, followed by longitudinal imaging from Day 8 to 11. IF staining and flow cytometry were performed on Day 8 and 11, respectively; (**b**) Photography of the affected paw on Day 0, 7 and 11. Foot swelling could be observed in the AIA group; (**c**) Measurements of paw thickness at different time points; (**d**) mRNA sequencing data were obtained from the right hind paw before and 8 days after CFA injection, and a comparison of log_2_(FPKM fold change) among different T-cell biomarkers is provided here. All the values represent the mean ± SEM unless otherwise specified. Unpaired 2-tailed Student’s *t* test was used for analyses, ****, *p* < 0.0001; ***, *p* < 0.001; **, *p* < 0.01; *, *p* < 0.05

To generate an AIA mouse model, CFA was injected into RP on Day 0 and Day 7 (Fig. 1a). Consistent with other studies [10], severe inflammation and foot swelling could be observed in RP in the AIA group (Fig. 1b). Paw thickness was measured with a calliper every day after the initial injection to monitor the severity of arthritis, and the paw thickness in the AIA group was greater than that in the control group at all time points (*P* < 0.001) (Fig. 1c). To further validate the inflammatory microenvironment in our AIA model, the expression of representative cytokines, such as IL-17, IL-1α, IL-1β and TNF-α, was measured via mRNA sequencing and ELISA. Both the mRNA and protein expression levels of all the cytokines tested were higher in the AIA cohort (Supplementary Fig. 1a and b). To identify potential candidate surface biomarkers for T-cell imaging, we analysed mRNA sequencing data from the RP before and 8 days after the initial CFA injection. We focused on certain T-cell activation markers, such as OX40, ICOS, CD69, 4-1BB, and GITR, for data analysis. Among all the biomarkers of interest, the 4-1BB gene was the most highly upregulated during the pathogenesis of arthritis (log2 FPKM fold change = 3.65; adjusted *p* value = 1.01E-020) (Fig. 1d).

### Analysis of 4-1BB expression during the pathogenesis of RA

**Fig. 2:**
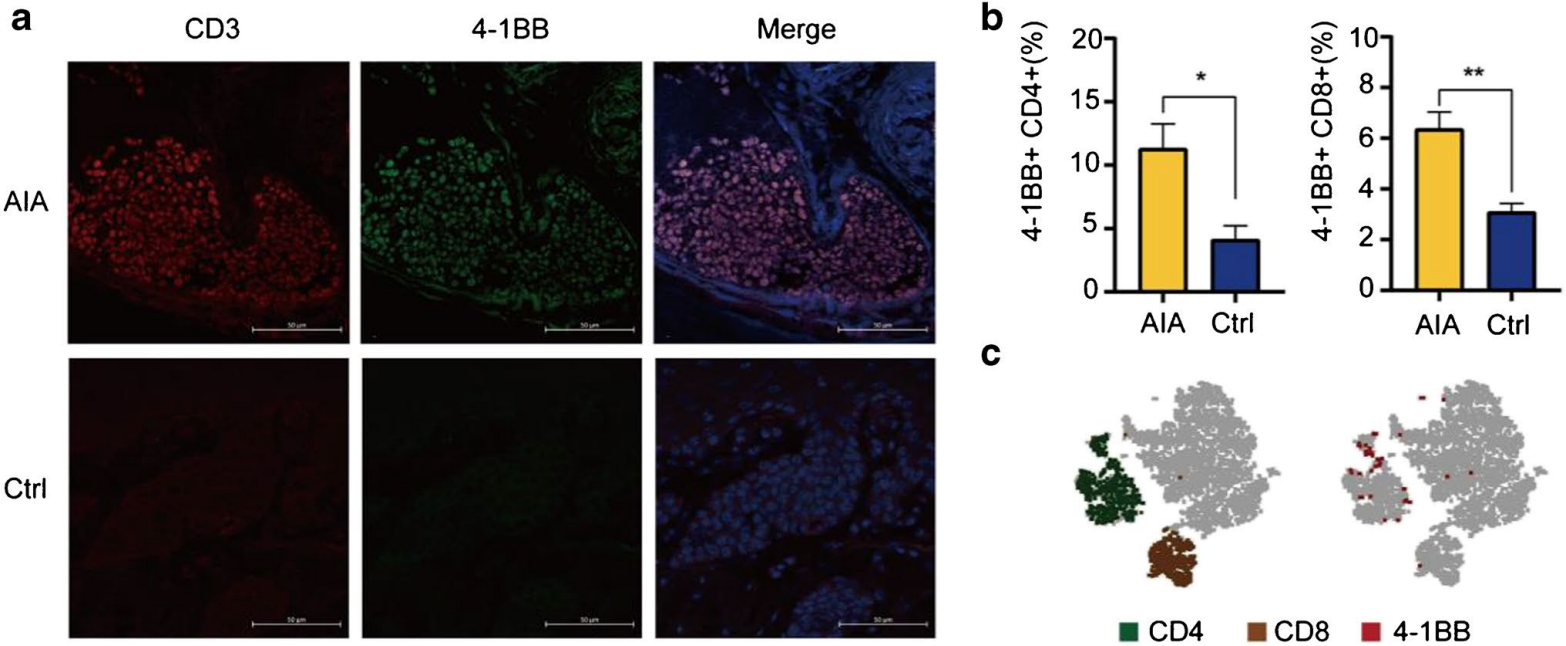
**a** IF staining was performed with the RP on day 8 after CFA injection, and colocalization could be observed between CD3 (red) and 4-1BB (green); (**b**) Comparison of 4-1BB expression between the AIA and control groups; (**c**) t-SNE plot analysis of 4-1BB expression on CD4^+^ and CD8.^+^ T cells. All the values represent the mean ± SEM unless otherwise specified. Unpaired 2-tailed Student’s *t* test was used for analyses, ****, *p* < 0.0001; ***, *p* < 0.001; **, *p* < 0.01; *, *p* < 0.05

Following the results of mRNA-seq, we analysed the kinetics of 4-1BB protein expression at different time points during the pathogenesis of RA. IF staining was performed with the RP, which was harvested on Day 8. Confocal imaging demonstrated massive CD3^+^ T cell infiltration (red panel) into the RP in the AIA group, and good colocalization of 4-1BB (green panel) and CD3 staining was also observed (Fig. 2a). On day 11, a separate group of mice was sacrificed for a flow cytometry study. As shown in Fig. 2b, 4-1BB expression on CD4^+^ and CD8^+^ T cells was higher in the AIA group (*P* < 0.05 and *P* < 0.01) than in the control groups. t-SNE analysis demonstrated that 4-1BB is mainly expressed on T-cell populations (Fig. 2c). Collectively, the mRNA-seq, flow cytometry, and IF staining data described above indicated the great potential of 4-1BB for use as a sensitive and specific imaging biomarker expressed on activated T cells during the pathogenesis of RA.

### IRDye-680RD-4-1BB mAb synthesis, characterization and cell uptake

**Fig. 3:**
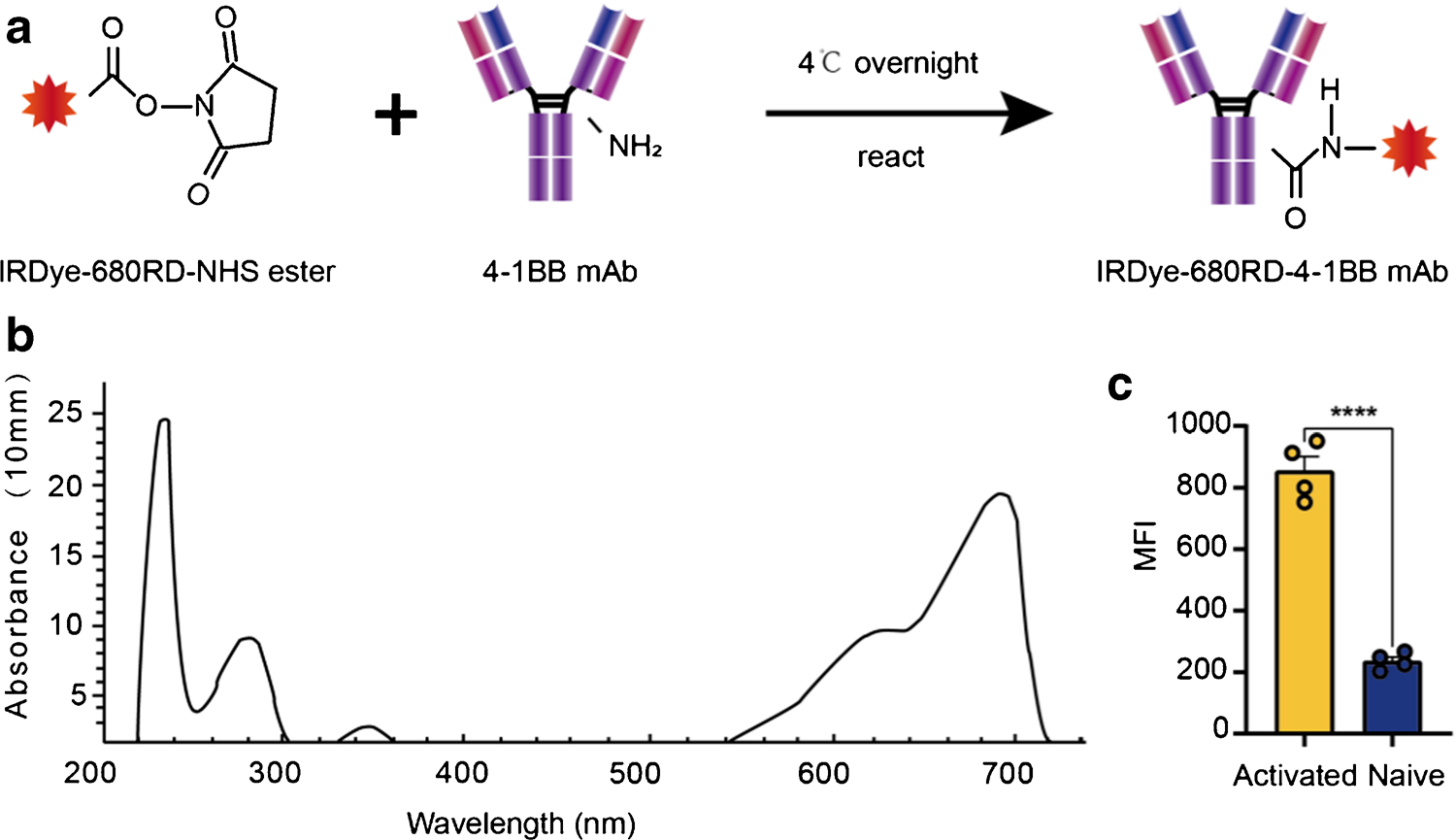
**a** Scheme: fluorophore conjugation of 4-1BB mAb with IRDye-680RD NHS ester; (**b**) Characterization of IRDye-680RD-4-1BB mAb by spectrum; (**c**) Cell binding assay to assess the specificity of IRDye-680RD-4-1BB mAb to activated T cells. All the values represent the mean ± SEM unless otherwise specified. Unpaired 2-tailed Student’s *t* test was used for analyses, ****, *p* < 0.0001; ***, *p* < 0.001; **, *p* < 0.01; *, *p* < 0.05

For IRDye-680RD-4-1BB mAb synthesis, the 4-1BB-specific mAb was incubated with IRDye-680RD-NHS ester at 4 °C overnight and then purified through a Vivaspin2 50 KDa cut-off spin filter (Fig. 3a). The probe absorbance and protein concentration were determined by Nanodrop (Fig. 3b). The chemical yield of the probe was 50–70% based on the calculation from 3 independent experiments. Further LC‒MS/MS analysis revealed that there were approximately three IRDye-680RD molecules chelated to one 4-1BB mAb on average, and the chemical purity of IRDye-680RD-4-1BB mAb was > 99% after purification (Supplementary Fig. 2). To validate the specificity of this probe for activated T cells, 2 μg of IRDye-680RD-4-1BB mAb was incubated with activated and naïve T cells at 37 °C for 1 h. After 3 washes, the mean fluorescence intensity (MFI) was determined via flow cytometry, and the MFI of the activated T-cell group was much higher than that of the naïve group, *P* < 0.0001 (Fig. 3c), which indicated the specificity of the IRDye-680RD-4-1BB mAb for activated T cells.

### In vivo NIRF imaging and ex vivo biodistribution

**Fig. 4:**
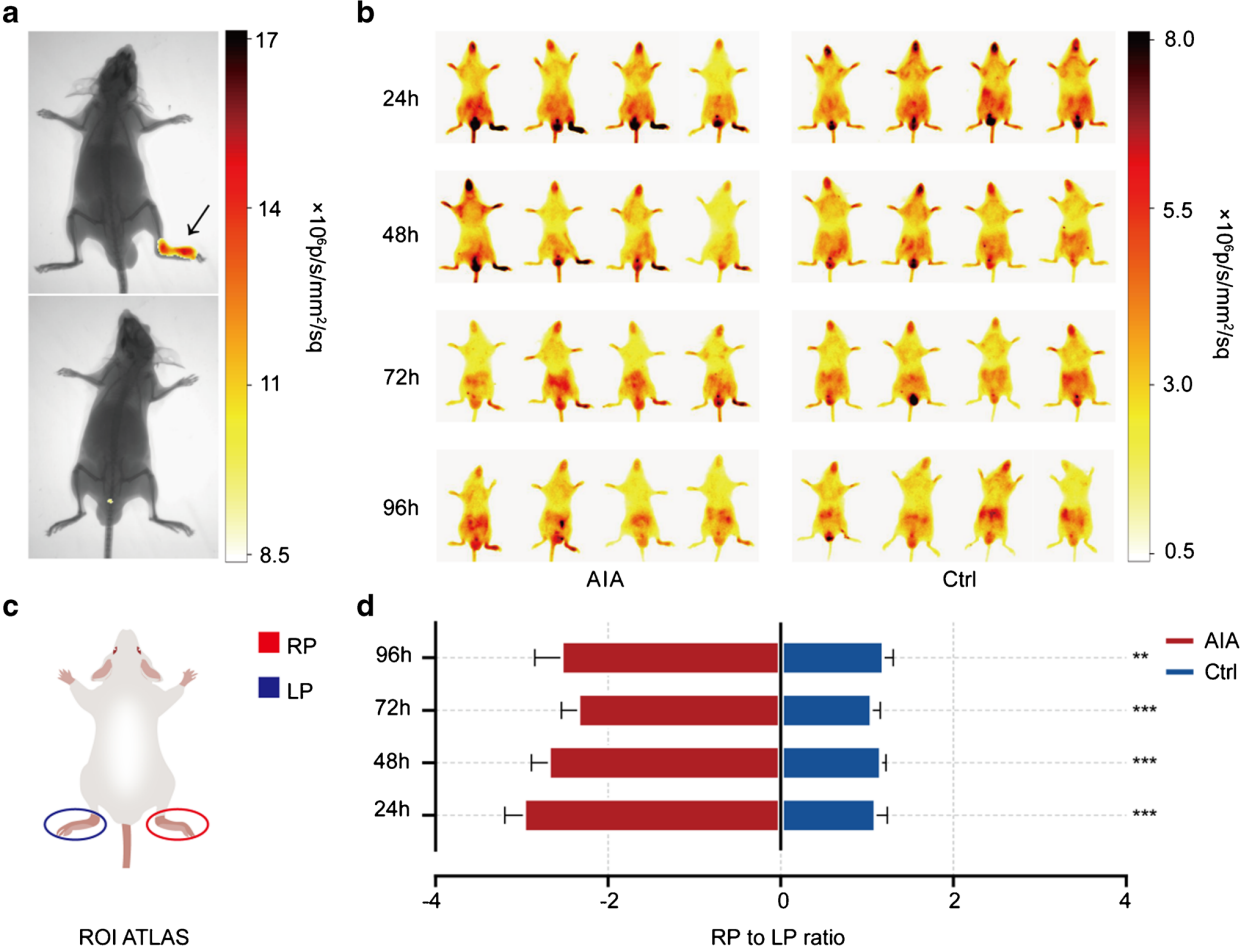
**a** X-ray and NIRF merged images at the 24-h time point, upper: AIA, lower: control, black arrow indicates CFA injected paw; (**b**) NIRF images at all time points between the AIA and control groups; (**c**) ROI atlas; (**d**) ROI quantification of the RP/LP ratio at all time points examined. All the values represent the mean ± SEM unless otherwise specified. Unpaired 2-tailed Student’s *t* test was used for analyses, ****, *p* < 0.0001; ***, *p* < 0.001; **, *p* < 0.01; *, *p* < 0.05

To evaluate the value of IRDye-680RD-4-1BB mAb in detecting 4-1BB^+^ activated T cells in vivo, 20 μg probe was injected into mice via the tail vein on Day 7. Longitudinal NIRF imaging was performed at 6, 24, 48, 72, and 96 h post-administration. As expected, an obvious accumulation of the optical probe was observed in RP of the AIA group at all time points examined (Supplementary Fig. 3a and Fig. 4a and b). ROIs were drawn to quantify the accumulation of the probe in the left hind paw (LP) and RP (Fig. 4c). The ratio of the fluorescence intensity of the RP and LP was used to compare the ROI profiles of the AIA and control groups. The RP/LP ratio in the AIA group was significantly higher than that in the control group (24 h: AIA = 2.99 ± 0.56, control = 1.11 ± 0.25; 48 h: AIA = 2.70 ± 0.50, control = 1.17 ± 0.09; 72 h: AIA = 2.36 ± 0.48, control = 1.06 ± 0.18; 96 h: AIA = 2.55 ± 0.78, control = 1.20 ± 0.19 and *P* value = 0.000148, 0.000227, 0.000644, 0.009087, respectively) (Fig. 4d). This observation may be attributed to the increased infiltration of 4-1BB^+^ activated T cells into the RP of the AIA group, which is also consistent with the flow cytometry and IF staining data described above.

**Fig. 5:**
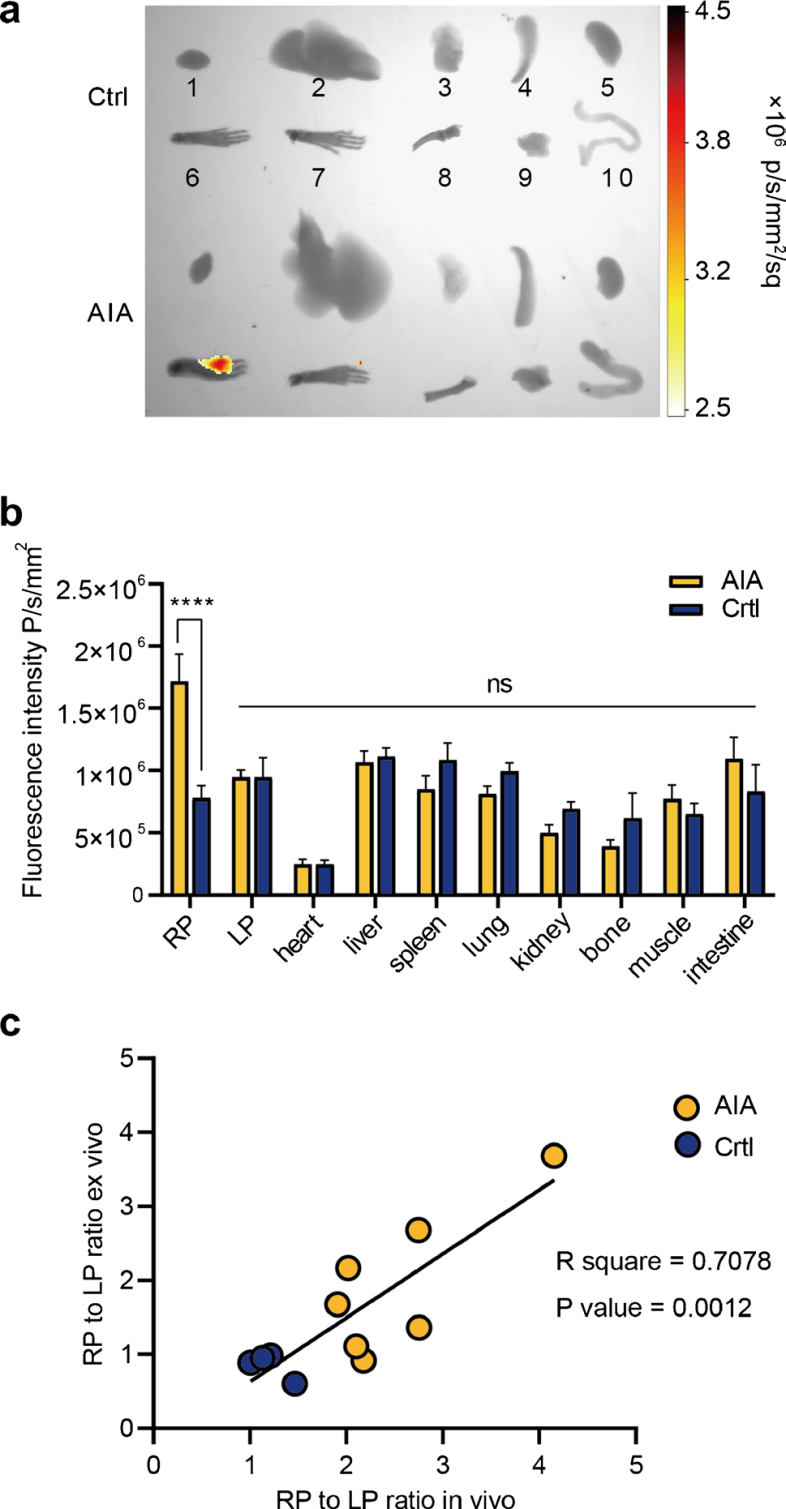
Immediately after the 96-h scan, certain organs (1. heart, 2. liver, 3. lung, 4. spleen, 5. kidney, 6. right hind paw, 7. left hind paw, 8. femur, 9. muscle, and 10. intestine) were harvested, ex vivo NIRF images were acquired (**a**), and semi-quantification was performed (**b**); (**c**). Correlation between in vivo RP/LP ratio and ex vivo RP/LP ratio. All the values represent the mean ± SEM unless otherwise specified. Unpaired 2-tailed Student’s *t* test was used for analyses, ****, *p* < 0.0001; ***, *p* < 0.001; **, *p* < 0.01; *, *p* < 0.05

To verify the ROI quantification, all the mice were euthanized immediately after the last scan. Certain organs were collected for ex vivo NIRF imaging (Supplementary Fig. 3b and Fig. 5a). A significant difference in the fluorescence intensity of the RP was observed (*P* < 0.05 and *P* < 0.0001, respectively), whereas no variation was observed in other organs (Supplementary Fig. 3c and Fig. 5b). Good concordance was also observed between the ROI results and the ex vivo biodistribution quantification results in terms of the RP/LP ratios (R square = 0.7078, *P* value = 0.0012) (Fig. 5c), which indicated the accuracy of the in vivo ROI measurements.

### Safety assessment and value of 4-1BB NIRF imaging in RA diagnosis

**Fig. 6:**
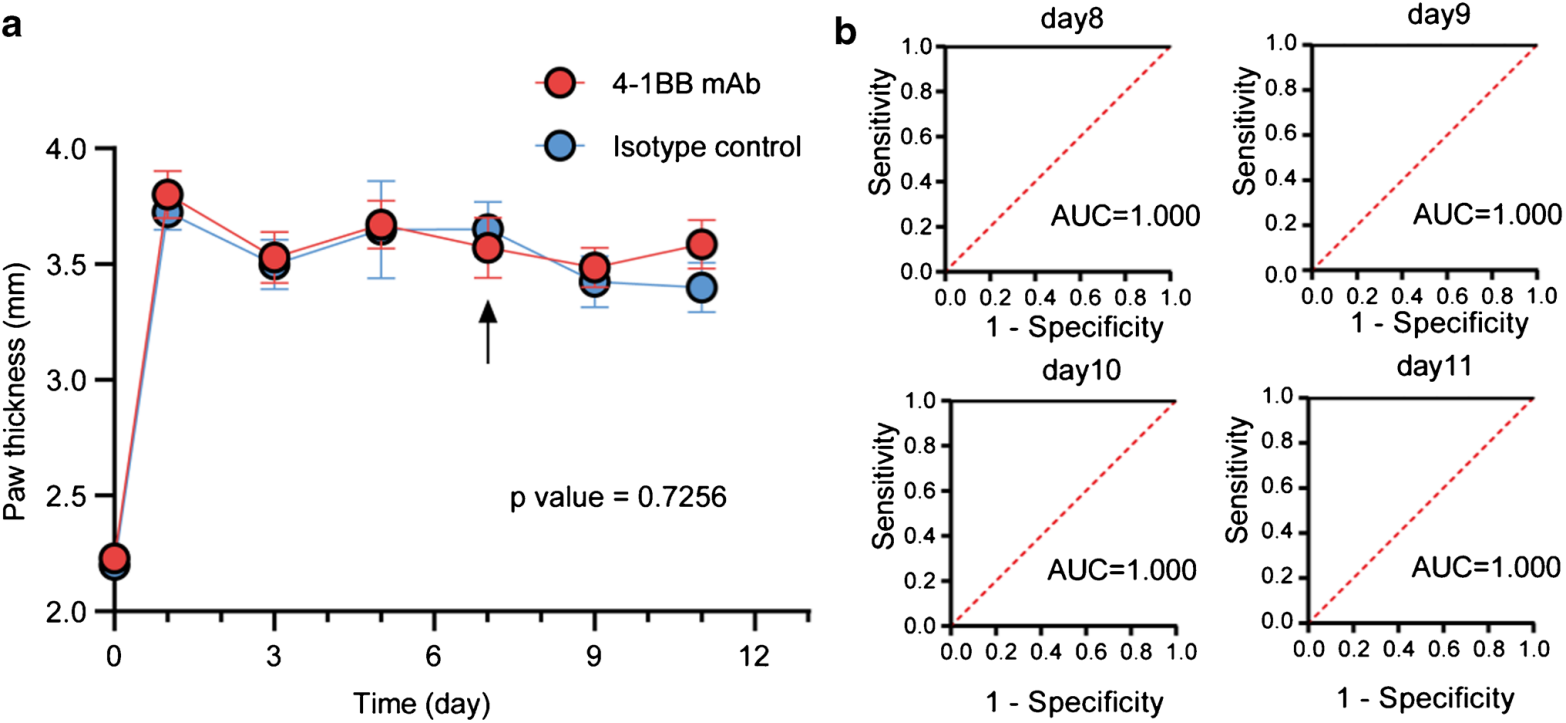
**a** Paw thickness measurements in AIA mice that received cold 4-1BB mAb or isotype control mAb; the *t* test was used to compare the difference between the two groups; the arrow indicates that the cold mAb was injected on Day 7; (**b**) ROC curve analysis showing sensitivity against 100% specificity for distinguishing the AIA group from the control group based on the RP/LP values at all time points imaged

Biosafety is crucial for molecular imaging probe development. As previously discussed [17], agonistic antibody-based probes may exacerbate inflammatory diseases. Thus, a separate group of AIA mice was injected with either 20 μg 4-1BB mAb (clone: 3H3) or 20 μg IgG2a isotype control (clone: 2A3), and no noticeable difference in paw thickness was observed between the two cohorts (Fig. 6a); these results indicate that certain dose of the probe does not exacerbate disease. To assess the diagnostic value of 4-1BB NIRF imaging in the early diagnosis of RA, we used receiver operating characteristic (ROC) analysis. RP/LP ROI quantification could perfectly differentiate the AIA group from the control group, which demonstrated the great potential of IRDye-680RD-4-1BB mAb NIRF imaging for RA diagnosis (Fig. 6b).

## Discussion

RA is an autoimmune disease that usually leads to joint disability, chronic pain, and even premature death. Early diagnosis and timely intervention are still the best ways to improve RA prognosis and lead to better quality of life [3]. Here, we demonstrated that imaging of activated T cells is a promising approach to achieve this goal, and 4-1BB could be an ideal biomarker for tracking activated T cells within the inflammatory environment during the pathogenesis of RA.

Since T-cell activation is considered as the initial event in many autoimmune diseases [18], the imaging of activated T-cell infiltration and distribution is theoretically the most sensitive method for assessing the pathogenesis of RA at the very beginning of the disease. As we discussed before [19], employing ‘omic’ data analysis could be a convenient and reliable tool for selecting candidate biomarkers for T-cell imaging. According to the mRNA-seq data obtained from the AIA model, many costimulatory molecules were upregulated in inflammatory arthritis, and 4-1BB was the most highly upregulated among all the genes of interest. Thus, 4-1BB was chosen. Notably, our mRNA sequencing data were consistent with previous studies that analysed 4-1BB expression in the RA model [20]. While coinhibitory molecules, such as PD-1 and CTLA-4, were also upregulated in the AIA model, this could be considered as the feedback mechanism to maintain immune homeostasis [21, 22]. Unlike the T-cell activation markers, the major function of coinhibitory molecules is to suppress T-cell function, and thus, these molecules cannot be used to image activated T cells; moreover, PD-1 and CTLA-4 expression is not restricted to T cells and are also expressed by B cells [23, 24]. Although some studies indicated that 4-1BB was also expressed by NK and DCs [25], our FACS t-SNE analysis demonstrated that 4-1BB expression was mainly restricted to activated T cells. IF staining showed co-expression of CD3 and 4-1BB in inflammatory arthritis tissues, which was also consistent with our FACS study. The data provided here indicated that the optical signal we captured via NIRF imaging should be highly attributed to activated T cells inside arthritic tissues. Thus, 4-1BB imaging outperforms other metabolic small molecule PET tracers (^18^F-FDG and ^18^F-AraG), which have low specificity for detecting activated T cells in such settings. Due to shallow penetration and intense light scattering, the optical imaging probe we used is suitable for superficial tissue/disease imaging, such as in RA and melanoma [26]. Moreover, NIRF imaging has other advantages, including non-radioactivity, easy preparation and low cost, which makes it more convenient for clinical translation. To our knowledge, this is the first study to identify 4-1BB as an imaging biomarker. With the synthesized NIRF probe, we could noninvasively visualize T-cell dynamics during the pathogenesis of RA in real time. The quantification of the ROI profiles could easily distinguish the AIA group from the control group at all time points examined, making 4-1BB imaging a reliable tool for RA diagnosis. With this promising approach, people could gain unique biological insights about T-cell responses and behaviour, which may help researchers and physicians better understand the mechanisms underlying certain diseases. This would definitely optimize RA patient management and therapeutic decision making.

The 4-1BB imaging work presented here does have limitations. The AIA mouse model used here exhibits only acute inflammatory arthritis [27, 28], and it does not model the chronic process of the pathogenesis of RA. Upon injection of CFA into the RP of mice, foot swelling appeared within 2 h or even less. Thus, it is impossible to acquire NIRF images before the appearance of RA symptoms in this model. To improve our work in the future, antigen-induced arthritis or collagen antibody-induced arthritis (CAIA) murine models, which mimic RA pathogenesis and the joint microenvironment, should be evaluated. Another limitation may be the use of a full-length mAb that includes a full Fc region, which may lead to nonspecific binding of the 4-1BB optical probe to Fc receptors expressed on other immune cells, such as macrophages, B cells, and DCs [29]. Like most mAbs imaging studies [30, 31], noticeable but inconsistent brain optical signals were observed in both AIA and control groups, which may be attributed to blood flow from murine mouth; thus, more efforts should be made to minimize the nonspecific binding and improve probe pharmacokinetics, such as developing novel protein scaffolds that lack Fc regions (scFv, fibronectin and nanobody). The diet of mice may also interfere with the imaging. Mice diets contain ingredients such as alfalfa, which produce background fluorescence at certain wavelengths (emission 740 nm); to reduce this false-positive signal, we are planning to use NIR-II (Near-infrared region-II) dye (1000–1700 nm) to improve the imaging quality in our next study.

Overall, in this proof-of-concept work, we first identified 4-1BB as a specific and conserved biomarker for visualizing activated T cells in inflammatory arthritis, and NIRF imaging enables noninvasive diagnosis of RA with high specificity and sensitivity in vivo. Given the promising results, we anticipate that the 4-1BB imaging strategy will have great potential for improving current approaches for RA diagnosis.

## Supplementary Information

Below is the link to the electronic supplementary material.

Supplementary file1 (PDF 130 KB)

Supplementary file2 (PDF 85 KB)

Supplementary file3 (PDF 168 KB)

## Data Availability

The authors declare that (the/all other) data supporting the findings of this study are available within article (and its supplementary information files).

## Code availability

The authors declare that there was no coding employed for data cleaning and analysis.
