# Supplementary material for: Fluorophore-conjugated 4-1BB antibody enables early detection of T-cell responses in inflammatory arthritis via NIRF imaging
Source: Eur J Nucl Med Mol Imaging. 2022 Sep 7;50(1):38–47. doi: 10.1007/s00259-022-05946-y (PMC9668804; doi:10.1007/s00259-022-05946-y)
Supplement: Supplementary file 1 — Supplementary file1 (PDF 130 KB) [file 259_2022_5946_MOESM1_ESM.pdf]

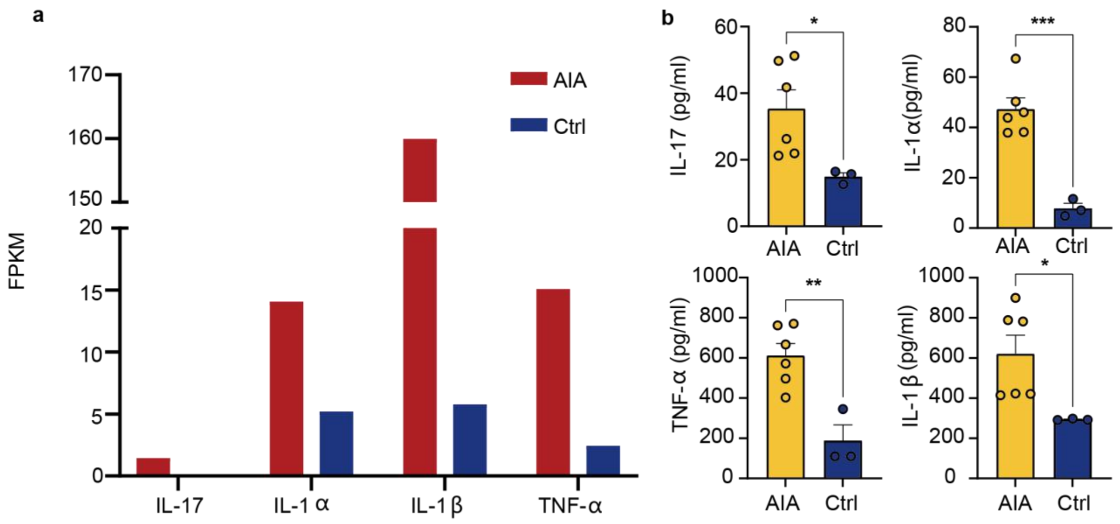

Supplementary Fig. 1 Enhanced expression of typical cytokines in the inflammatory arthritis model. **(a)** Comparison of FPKM (fragments per kilobase of exon model per million mapped fragments) of IL-17, IL-1α, IL-1β, and TNF-α between the AIA and control groups on Day 8; **(b)** The concentrations of IL-17, IL-1α, IL-1β and TNF-α were measured by ELISA.All the values represent the mean ± SEM unless otherwise specified. Unpaired 2-tailed Student's t testwas used for analyses, ****, $p < 0.0001$; ***, $p < 0.001$; **, $p < 0.01$; *, $p < 0.05$.
