# Supplementary material for: Fluorophore-conjugated 4-1BB antibody enables early detection of T-cell responses in inflammatory arthritis via NIRF imaging
Source: Eur J Nucl Med Mol Imaging. 2022 Sep 7;50(1):38–47. doi: 10.1007/s00259-022-05946-y (PMC9668804; doi:10.1007/s00259-022-05946-y)
Supplement: Supplementary file 2 — Supplementary file2 (PDF 85 KB) [file 259_2022_5946_MOESM2_ESM.pdf]

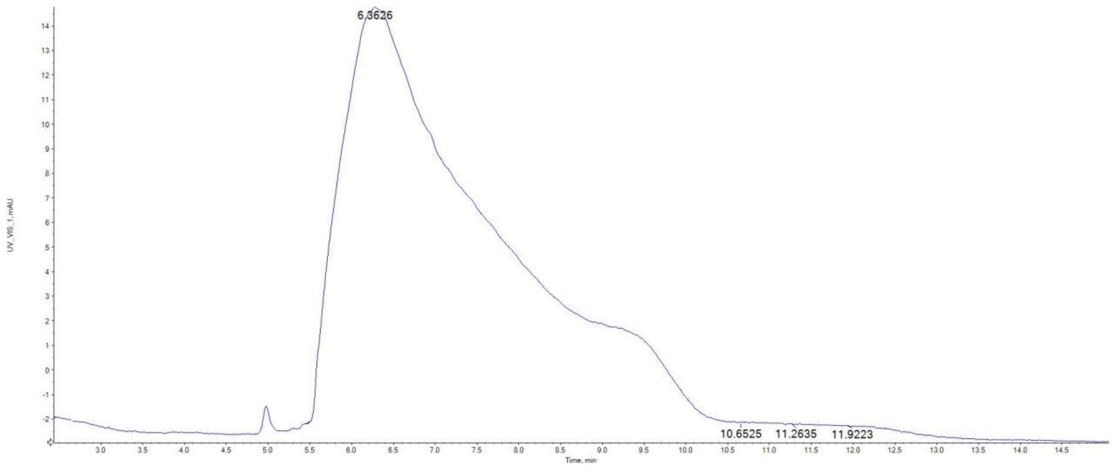

Supplementary Fig. 2 The chemical purity of the IRDye-680RD-4-1BB mAb was >99% after purification via a Vivaspin2 50 kDa cut-off spin filter.
