# Supplementary material for: Fluorophore-conjugated 4-1BB antibody enables early detection of T-cell responses in inflammatory arthritis via NIRF imaging
Source: Eur J Nucl Med Mol Imaging. 2022 Sep 7;50(1):38–47. doi: 10.1007/s00259-022-05946-y (PMC9668804; doi:10.1007/s00259-022-05946-y)
Supplement: Supplementary file 3 — Supplementary file3 (PDF 168 KB) [file 259_2022_5946_MOESM3_ESM.pdf]

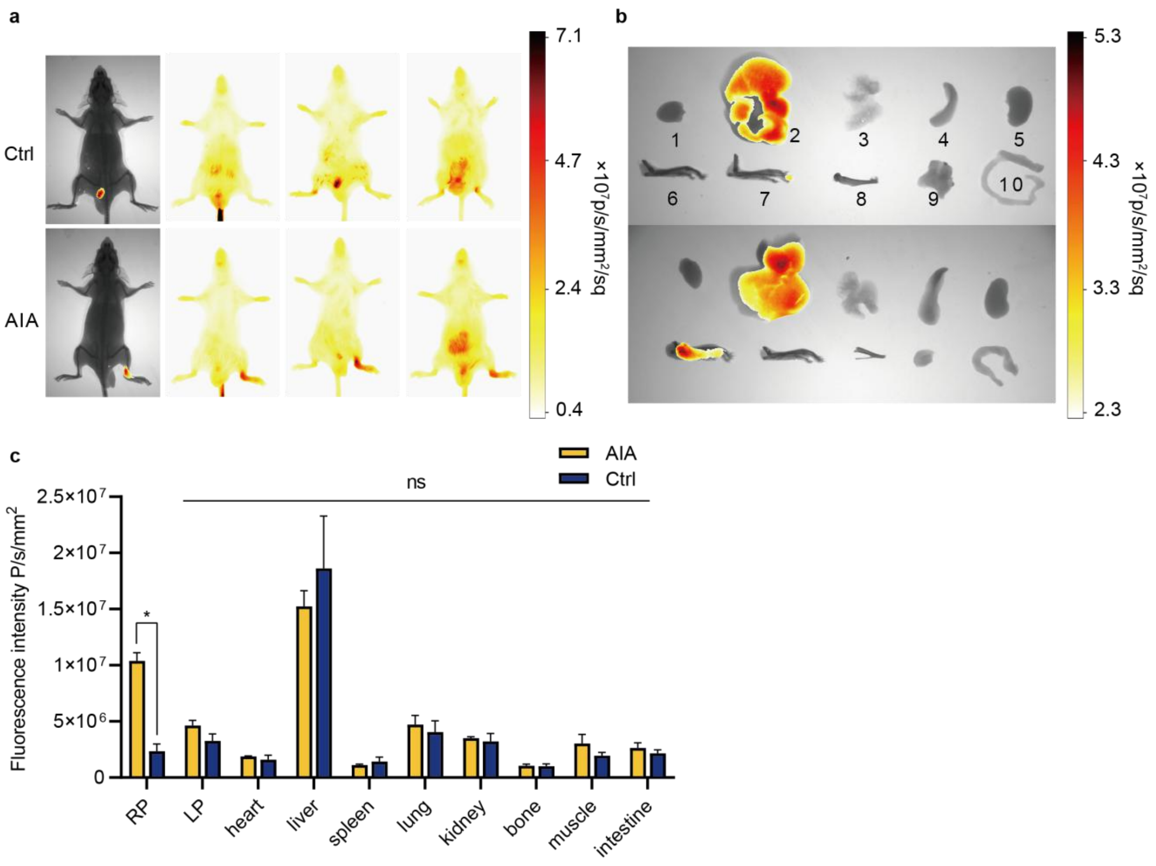

Supplementary Fig. 3 NIRF imaging of AIA (n= 3) and Ctrl mice (n= 3). **(a)** X-ray and NIRF merged images 6 hours after the injection of IRDye680RD-4-1BB mAb.; **(b)**. Immediately after the 6-hour scan, certain organs (1. heart, 2. liver, 3. lung, 4. spleen, 5. kidney, 6. right hind paw, 7. left hind paw, 8. femur, 9. muscle, and 10. intestine) were collected and ex vivo NIRF images were acquired. **(c)** Semi-quantification was counted. All values represent the mean ± SEM unless otherwise specified. Unpaired 2-tailed Student's t test was used for analyses, ****, $p < 0.0001$; ***, $p < 0.001$; **, $p < 0.01$; *, $p < 0.05$.
